# Supplementary material for: Technical Evaluation of a New Medical Device Based on Rigenase in the Treatment of Chronic Skin Lesions
Source: Bioengineering (Basel). 2023 Aug 29;10(9):1022. doi: 10.3390/bioengineering10091022 (PMC10526047; doi:10.3390/bioengineering10091022)
Supplement: Supplementary file 1 [file bioengineering-10-01022-s001.zip › bioengineering-2567658-supplementary.pdf]

SUPPLEMENTARY DATA

Original Research

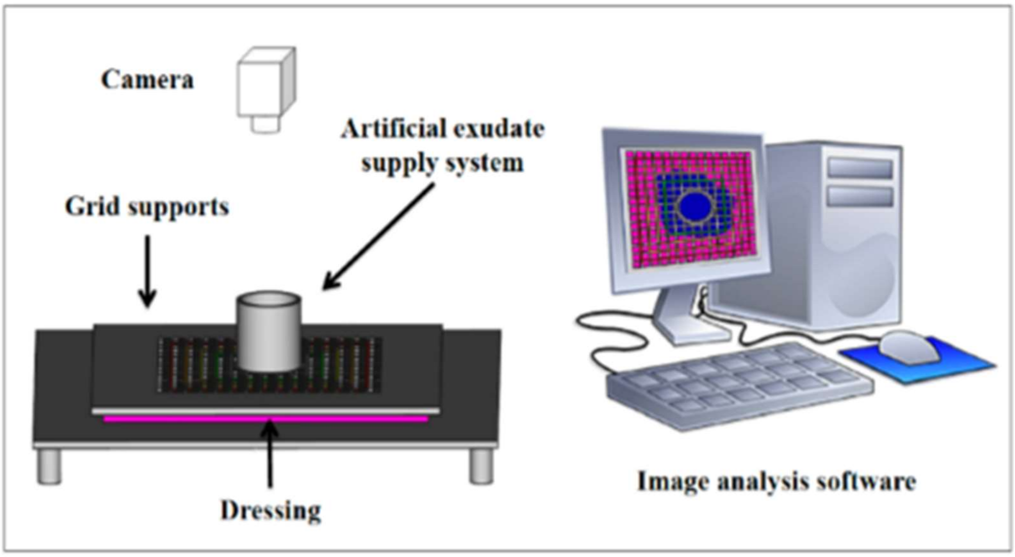

Supp Fig. S1 Representation of the device reproducing the clinical management of the wound dressing in vitro.

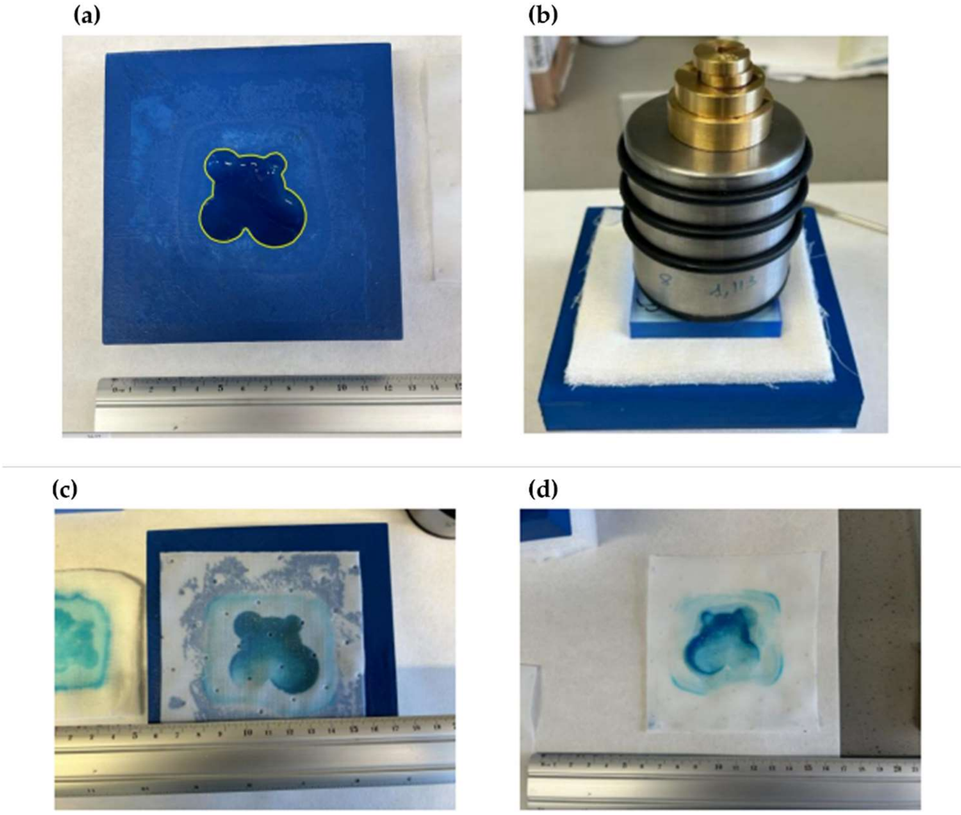

Supp Fig. S2 In vitro simulation of elastocompressive bandage and dressing of a chronic wound with abundant exudate production. **(a)** Simulated Lesion **(b)** lateral diffusion test simulating elastic compression **(c)**External Lateral Diffusion **(d)** Internal Lateral Diffusion.
